# Defensive medicine–related practices among child and adolescent psychiatrists in Türkiye: prevalence, behavioral patterns, and associated factors

**DOI:** 10.3389/fpsyt.2026.1860742

**Published:** 2026-09-22

**Authors:** Esra Hoşoğlu, Bedia Sultan Önal, Bahadir Turan

**Affiliations:** 1 Faculty of Medicine, Karadeniz Technical University, Trabzon, Türkiye; 2 Department of Child and Adolescent Psychiatry, Giresun Universitesi Tip Fakultesi, Giresun, Türkiye

**Keywords:** child and adolescent psychiatry, clinical decision-making, defensive medicine, defensive practice, malpractice

## Abstract

**Introduction:**

Defensive medicine (DM) refers to practices adopted to avoid potentially distressing medico-legal situations. Although DM has been widely studied across various medical specialties, evidence in psychiatry remains limited, and no studies have specifically examined this issue in child and adolescent psychiatry. This study aimed to examine the prevalence of DM among child and adolescent psychiatrists in Türkiye and to identify associated factors.

**Methods:**

This cross-sectional study was conducted using an anonymous online survey distributed between November 2024 and February 2025. The survey included a sociodemographic and professional data form and a questionnaire assessing DM-related practices. Participants who reported engaging in defensive practices in at least half of their patients were classified as practicing DM. Group comparisons were conducted using appropriate statistical methods, and logistic regression analysis was performed to identify independent predictors.

**Results:**

A total of 203 child and adolescent psychiatrists participated, of whom 41.4% were classified as practicing DM. No significant differences were observed between groups in terms of sociodemographic or professional characteristics. DM-related practices were commonly reported across multiple domains, including patient assessment, pharmacological decision-making, suicide risk management, and report-related practices. Psychiatrists who reported practicing DM demonstrated significantly higher frequencies of several behaviors such as not initiating assessment or pharmacotherapy in the absence of a parent or legal guardian, increased documentation and detailed disclosure of rare adverse effects, and cardiology referrals before initiating stimulant or atomoxetine treatment without clear indication. In regression analysis, a higher number of health committee patients (OR = 2.198, p = 0.017), detailed disclosure of rare but severe adverse effects (OR = 1.580, p = 0.002), and cardiology referral without clinical indication (OR = 1.419, p = 0.010) were identified as significant predictors of DM.

**Conclusion:**

DM-related practices appear to be common among child and adolescent psychiatrists in Türkiye and may reflect both medico-legal concerns and context-specific features of clinical practice. Such practices are more closely associated with specific clinical situations than with sociodemographic or professional characteristics. These findings underscore the need for further research and for context-sensitive strategies to reduce defensive practices while maintaining high-quality patient care.

## 1 Introduction

The increasing number of malpractice lawsuits against physicians, the ease of filing complaints through various channels, and the rising incidence of violence toward healthcare professionals have recently led physicians to adopt self-protective behaviors (1). Defensive medicine (DM) refers to the attitudes and practices physicians adopt during or after clinical care to avoid potentially distressing situations, such as being sued or reported by patients (2). These behaviors, while aimed at reducing legal exposure, may influence clinical decision-making and patient care (3, 4).

Beyond its implications for individual clinical decision-making, DM may also have broader implications at the health system level, particularly with regard to economic burden and resource utilization. In this context, DM has been associated with increased healthcare expenditures and the use of unnecessary, non-evidence-based interventions, which may in turn expose patients to avoidable risks (2). A nationwide survey conducted in Italy involving over 1,300 physicians concluded that DM may represent up to 10% of national health expenditures (5). In a study from the U.S. examining over 4,000 procedures across three hospitals, 28% of the interventions were identified as defensive, with an estimated 13% of patient-level costs attributed to such practices (6). In light of these economic and clinical consequences, examining the extent and evolving prevalence of DM across medical specialties may help to better contextualize its broader impact.

Recent studies have reported an increase in the practice of DM among physicians. In a multinational study of infectious disease and clinical microbiology physicians from 74 countries, 85.0% of participants reported engaging in defensive behaviors when prescribing antibiotics (7). Similarly, in a study conducted among physicians in the surgical departments of a Turkish university hospital, 94.2% reported engaging in at least one form of defensive medicine, with the highest scores observed in pediatric surgery (8). Supporting these findings, a recent meta-analysis that included 64 studies involving 35,900 physicians from 23 countries reported a pooled global prevalence of defensive medicine at 75.8%, with the highest rates observed among anesthesiologists (92.2%), physicians in the African region (88.1%), and those in lower-middle-income countries (89.0%) (9). These consistent findings across various medical settings underscore the widespread nature of DM and the urgent need for targeted strategies to address its underlying causes.

Although psychiatry shares certain clinical features with other medical specialties, such as careful prescribing, systematic follow-up, and thorough documentation, it is characterized by distinctive challenges inherent to mental healthcare. Psychiatrists frequently manage high-risk situations, including suicide risk assessment, decisions regarding involuntary hospitalization, and the treatment of forensic patients (10–12). Existing literature suggests that DM is also prevalent within psychiatric practice, with younger clinicians and those with prior exposure to malpractice litigation appearing more likely to engage in defensive behaviors (2, 11, 13). Within this broader psychiatric context, child and adolescent psychiatry entails additional clinical, ethical, and legal complexities. Clinicians must account for the developmental status of minors, their evolving decision-making capacity, and their dependence on parents or guardians. Balancing respect for a minor’s emerging autonomy with protective responsibilities, navigating confidentiality within family systems, and determining treatment in the child’s best interests create a particularly sensitive practice environment (14, 15). These layered vulnerabilities and ethical tensions may further shape defensive attitudes and practices in this field, underscoring the relevance of specifically examining DM within child and adolescent psychiatry.

These clinical and ethical challenges are further compounded by the medico-legal responsibilities inherent to child and adolescent psychiatric practice (14, 15). In Türkiye, child and adolescent psychiatrists practice within a legal and regulatory framework that requires them to balance legal obligations, ethical principles, and the developmental needs of minors in everyday clinical practice. Their responsibilities extend beyond diagnosis and treatment to encompass mandatory reporting obligations, informed consent procedures involving minors and their parents or legal guardians, and the preparation of disability and other medico-legal reports that carry important legal, educational, and administrative consequences (16–18). Previous studies from Türkiye have reported that child and adolescent psychiatrists experience difficulties during disability evaluations because these assessments may substantially influence children’s educational opportunities, access to services, and legal rights (17, 19). Similarly, mandatory reporting obligations, particularly in cases of suspected child abuse and neglect, may require clinicians to balance patient confidentiality with legal duties and the child’s best interests, while treatment decisions frequently require balancing parental authority with the evolving autonomy and decision-making capacity of minors (15, 16, 20).

These medico-legal responsibilities may become particularly challenging when legal obligations, ethical principles, and clinical judgment do not always align. Furthermore, routine clinical ethics consultation services are currently unavailable for day-to-day clinical practice in Türkiye, requiring physicians to rely primarily on their own ethical judgment and legal responsibilities when managing ethically complex situations (16). Psychiatric diagnoses can carry long-term social and occupational implications that extend beyond the immediate clinical encounter (15, 21, 22). In Türkiye, psychiatric diagnoses or mental health conditions may, in some cases, pose barriers to eligibility for certain professions, such as police or military service (23, 24). Consequently, clinicians may receive requests from patients or families to modify or remove psychiatric diagnoses from medical records because of concerns regarding future occupational opportunities. In some instances, these requests may place clinicians under considerable professional or medico-legal pressure (25). Collectively, these circumstances may increase concerns regarding potential complaints or legal consequences and encourage more cautious or defensive clinical practices among child and adolescent psychiatrists.

Given these unique dynamics and the high-stakes nature of clinical decision-making in this population, it is essential to examine how defensive medical practices manifest in child and adolescent psychiatry and to what extent they may be shaped by these distinctive ethical and legal considerations. To the best of our knowledge, no study has specifically and comprehensively evaluated the defensive approaches of child and adolescent psychiatrists. This study therefore aims to fill this gap by evaluating the defensive medical approaches of child and adolescent psychiatrists in Türkiye.

## 2 Materials and methods

This cross-sectional survey was conducted between November 2024 and February 2025. A link to an anonymous survey was designed using “Google Forms” (Google LLC, Mountain View, CA, USA). The survey was initially distributed through professional WhatsApp groups whose membership was restricted to child and adolescent psychiatry specialists, academic faculty members, and residents across Türkiye. In addition, the survey link was shared with eligible colleagues through the researchers’ professional networks, with further dissemination to other eligible child and adolescent psychiatrists through these networks. Thus, the recruitment strategy incorporated elements of snowball sampling. Because the exact number of eligible physicians who ultimately received the survey invitation could not be determined, a response rate could not be calculated. For contextual reference, current membership data were subsequently obtained from the Turkish Association for Child and Adolescent Psychiatry, the national professional association for child and adolescent psychiatry in Türkiye. As of August 20, 2026, the Association reported 1,824 physician members (740 residents, 781 specialists, and 303 academic faculty members) (26). As these data postdated the study period and did not represent the number of physicians who received the survey invitation, they were not used to calculate a response rate.

At the beginning of the survey, participants were provided with an explanatory text describing the study and defining defensive medicine as attitudes and behaviors adopted by physicians to avoid adverse consequences such as complaints, disciplinary actions, legal proceedings, or media exposure related to their professional practice. The distinction between positive and negative defensive medicine was also explained. The introductory text provided to participants is presented in **Supplementary 1**. Participants were informed that participation was entirely voluntary, and completion of the survey was considered provision of informed consent.

The survey consisted of two main parts:

### 2.1 Sociodemographic and professional data form

A data form developed by the researchers was used to collect information regarding the participants. The form included sociodemographic characteristics (such as age, gender, marital status, and number of children) as well as professional variables (such as type of institution, academic title, years of experience, number of patients seen daily, frequency of on-call duties, and exposure to malpractice or formal reporting processes).

### 2.2 Defensive medicine–related practices

The survey included items designed to explore defensive medicine–related practices among child and adolescent psychiatrists. Because no validated questionnaire specifically addressing defensive medicine in this population was available, the items were developed by the researchers with reference to previous studies of defensive medicine in psychiatry (2, 13, 27). An initial set of items was prepared by one child and adolescent psychiatrist based on previous defensive medicine literature and clinical experience, incorporating previously described defensive behaviors where applicable as well as specialty-specific situations involving ethical, legal, and medico-legal considerations commonly encountered in child and adolescent psychiatric practice. The items were subsequently reviewed by two other child and adolescent psychiatrists, who suggested additional items before the final set was established.

The questions addressed four thematic areas: patient assessment, initiation or modification of pharmacological treatment, management of patients with suicidal ideation, and forensic and legal reporting practices. Each item was rated on a 5-point Likert scale ranging from 1 (“in no patient”) to 5 (“in every patient”), as detailed in **Supplementary Material 1**. The items were selected to assess clinical practices that may occur in the context of defensive medicine, as defined to participants at the beginning of the survey.

Participants were asked to indicate, using a 5-point Likert scale, the proportion of patients in whom they practiced defensive medicine in their daily clinical work. Participants who self-reported practicing defensive medicine in at least half of their patients were categorized as the DM group. This classification was based on the approach used by Reuveni et al. in a survey of psychiatrists and was subsequently adopted in later studies investigating defensive medicine among psychiatrists (2, 11).

The study received ethical approval from the Clinical Research Ethics Committee of Giresun Training and Research Hospital (Approval No: BAEK-109; Decision Date: 18.09.2024; Decision No: 18.09.2024/07). The study was conducted in accordance with the ethical standards of the institutional research committee and with the 1964 Helsinki Declaration and its later amendments or comparable ethical standards.

### 2.3 Statistical analysis

All statistical analyses were performed using IBM SPSS Statistics version 22.0 (SPSS Inc., Chicago, IL, USA). Descriptive statistics (means, standard deviations, frequencies, and percentages) were calculated to summarize the sociodemographic and professional characteristics of the participants. The normality of data distribution was assessed using the Kolmogorov–Smirnov test. Group comparisons between participants classified as the DM and non-DM groups according to the predefined self-reported frequency criterion were performed using independent samples t-tests for normally distributed continuous variables and Mann–Whitney U tests for non-normally distributed variables. Categorical variables were analyzed using chi-square tests.

To examine differences in defensive medicine–related practices, responses to individual items were compared between the DM and non-DM groups. Mean scores and standard deviations were reported, and statistical significance was determined using t-tests or Mann–Whitney U tests based on the distribution of each item.

Additionally, binary logistic regression analyses were conducted to identify significant predictors of self-reported DM practice. Variables that were found to be associated with DM practice at a significance level of p < 0.10 in univariate analyses were included in the initial model. A backward stepwise elimination method was then applied to obtain the final model. For regression analyses, selected categorical variables were regrouped into broader categories where appropriate to avoid sparse or highly unbalanced categories. Odds ratios (ORs) and 95% confidence intervals (CIs) were calculated for each independent variable. A p-value of less than 0.05 was considered statistically significant.

## 3 Results

### 3.1 Sample characteristics

The sample consisted of 203 child and adolescent psychiatrists. The majority of participants were between 31 and 40 years of age (62.0%), female (80.8%), and married (70.9%). Most physicians were employed at university hospitals (31.0%) or training and research hospitals (29.1%). In terms of academic position, the largest group consisted of specialists (57.1%), followed by residents (26.6%), with a small proportion holding professorial titles. Only a small proportion of participants (1.0%, n = 2) reported personal involvement in a malpractice lawsuit, while 15.8% (n = 32) indicated that someone close to them had been involved in such a case. Half of the physicians (50.2%, n = 102) reported having been the subject of a formal complaint through official channels, and 76.8% (n = 156) stated that someone close to them had faced such a complaint (**Supplementary Material 2**).

According to the criteria defined in the Method section for identifying physicians who practice DM, 84 participants (41.4%) were classified as the DM group, while 119 (58.6%) were assigned to the non-DM group.

### 3.2 Comparison of sociodemographic and professional characteristics between DM and non-DM groups

The comparison of sociodemographic and professional characteristics between the DM and non-DM groups revealed no statistically significant differences across variables such as age, gender, marital status, years of experience, institutional affiliation, academic title, workload, perceived competence, or prior exposure to malpractice processes. Although the DM group reported a higher weekly patient evaluation in forensic and committee settings, these differences did not reach statistical significance (p = 0.056 and p = 0.052, respectively) (**Table 1**).

**Table 1:** Group differences in sociodemographic and professional variables.

Characteristics	Category	DM group n:84 (41.4%)	No-DM group n:119 (58.6%)	χ^2^	df	p
Age (years)	25–30	23 (27.4)	42 (35.3)	5.944	3	0.114
	31–40	58 (69.0)	68 (57.1)			
	41–50	2 (2.4)	9 (7.6)			
	>50	1 (1.2)	0 (0.0)			
Gender	Female	66 (78.6)	98 (82.4)	0.454	1	0.501
	Male	18 (21.4)	21 (17.6)			
Marital status	Married	60 (71.4)	84 (70.6)	0.170	1	0.897
	Single	24 (28.6)	35 (29.4)			
Having children	Yes	34 (40.5)	49 (41.2)	0.100	1	0.920
	No	50 (59.5)	70 (58.8)			
Institution of employment	State hospital	15 (17.9)	17 (14.3)	6.885	5	0.229
	Training and research hospital	29 (34.5)	30 (25.2)			
	City hospital	8 (9.5)	15 (12.6)			
	University hospital	25 (29.8)	38 (31.9)			
	Private hospital	1 (1.2)	0 (0.0)			
	Private clinic	6 (7.1)	19 (16.0)			
Department position	Professor	0 (0.0)	1 (0.8)	3.126	4	0.537
	Associate Professor	7 (8.3)	5 (4.2)			
	Assistant Professor	9 (10.7)	11 (9.2)			
	Specialist	49 (58.3)	67 (56.3)			
	Resident	19 (22.6)	35 (29.4)			
Years of experience as a physician (years)	0–5	19 (22.6)	37 (31.1)	5.178	4	0.270
	6–10	41 (48.8)	41 (34.5)			
	11–15	20 (23.8)	30 (25.2)			
	16–20	3 (3.6)	8 (6.7)			
	>20	1 (1.2)	3 (2.5)			
Years of experience in child and adolescent psychiatry (years)						
	0–5	29 (34.5)	51 (43.2)	5.054	4	0.282
	6–10	43 (51.2)	42 (35.6)			
	11–15	9 (10.7)	19 (16.1)			
	16–20	2 (2.4)	4 (3.4)			
	>20	1 (1.2)	2 (1.7)			
Monthly on-call duties in the past year	0	23 (27.4)	44 (37.0)	2.773	3	0.428
	<5	20 (23.8)	29 (24.4)			
	5–10	23 (27.4)	28 (23.5)			
	>10	18 (21.4)	18 (15.1)			
Number of patients seen daily	<10	15 (17.9)	33 (27.7)	4.255	3	0.235
	10–20	30 (35.7)	29 (24.4)			
	21–30	31 (36.9)	46 (38.7)			
	>30	8 (9.5)	11 (9.2)			
Weekly number of health committee patients	0	11 (13.1)	32 (26.9)	9.415	4	0.052
	<10	22 (26.2)	36 (30.3)			
	10–20	22 (26.2)	24 (20.2)			
	21–30	13 (15.5)	8 (6.7)			
	>30	16 (19.0)	19 (16.0)			
Weekly number of forensic patients	0	18 (21.4)	40 (33.6)	7.574	3	0.056
	<5	43 (51.2)	62 (52.1)			
	5–10	21 (25.0)	14 (11.8)			
	>10	2 (2.4)	3 (2.5)			
Is there a child psychiatry inpatient unit at your institution?						
	Yes	16 (19.0)	23 (19.3)	0.002	1	0.960
	No	68 (81.0)	96 (80.7)			
Perceived professional competence	Very low	0 (0.0)	0 (0.0)	1.389	3	0.708
	Low	4 (4.8)	7 (5.9)			
	Moderate	20 (23.8)	33 (27.7)			
	Good	50 (59.5)	61 (51.3)			
	Very good	10 (11.9)	18 (15.1)			
Level of professional satisfaction	Very low	1 (1.2)	2 (1.7)	3.785	4	0.436
	Low	5 (6.0)	4 (3.4)			
	Moderate	20 (23.8)	27 (22.7)			
	Good	43 (51.2)	52 (43.7)			
	Very good	15 (17.9)	34 (28.6)			
Involved in a malpractice lawsuit?	Yes	2 (2.4)	0 (0.0)	2.862	1	0.091
	No	82 (97.6)	119 (100.0)			
Anyone involved in a malpractice lawsuit?	Yes	16 (19.0)	16 (13.4)	1.164	1	0.281
	No	68 (81.0)	103 (86.6)			
Reported via formal channels?	Yes	46 (54.8)	56 (47.1)	1.169	1	0.280
	No	38 (45.2)	63 (52.9)			
Anyone close reported via formal channels?	Yes	67 (79.8)	89 (74.8)	0.684	1	0.408
	No	17 (20.2)	30 (25.2)			

### 3.3 Distribution of responses to the defensive medicine–related practice items

Participant responses to the defensive medicine–related practice items varied across the clinical situations assessed (**Table 2**). Specifically, 46.3% reported that they did not initiate clinical assessments without the presence of a parent or legal guardian in every patient, while 59.6% applied the same approach to psychotherapy. Additionally, 77.8% indicated that they did not prescribe medication without a parent or guardian present.

**Table 2:** Distribution of participant responses to the defensive medicine–related practice items.

Practice item	1 (In no patient) N (%)	2 (In a few patients) N (%)	3 (In half of the patients) N (%)	4 (In most patients) N (%)	5 (In every patient) N (%)
Patient assessment					
No assessment without parent or legal guardian	12 (5.9)	20 (9.9)	8 (3.9)	69 (34.0)	94 (46.3)
No psychotherapy without parent or legal guardian	15 (7.4)	11 (5.4)	8 (3.9)	48 (23.6)	121 (59.6)
No pharmacotherapy without parent or legal guardian	18 (8.9)	2 (1.0)	2 (1.0)	23 (11.3)	158 (77.8)
Medication not prescribed without patient	26 (12.8)	27 (13.3)	36 (17.7)	45 (22.2)	69 (34.0)
Diagnostic coding contingent on treatment decision	18 (8.9)	23 (11.3)	41 (20.2)	55 (27.1)	66 (32.5)
Confirmatory tests sought despite clear clinical impression	6 (3.0)	33 (16.3)	46 (22.7)	76 (37.4)	42 (20.7)
Patients medication					
Inform about rare but severe adverse effects	8 (3.9)	36 (17.7)	45 (22.2)	63 (31.0)	51 (25.1)
Record that explained about side effects	17 (8.4)	40 (19.7)	31 (15.3)	42 (20.7)	73 (36.0)
Obtain signed consent outlining side effects	164 (80.8)	29 (14.3)	5 (2.5)	3 (1.5)	2 (1.0)
Inform about suicidality associated with SSRI treatment	52 (25.6)	64 (31.5)	49 (24.1)	29 (14.3)	9 (4.4)
Routine labs ordered in absence of recent results	9 (4.4)	24 (11.8)	33 (16.3)	59 (29.1)	78 (38.4)
Referral to cardiology despite absence of indication before mph/atx treatment	71 (35.0)	71 (35.0)	27 (13.3)	12 (5.9)	22 (10.8)
Inform about special (regulated) prescription for mph	11 (5.4)	28 (13.8)	28 (13.8)	35 (17.2)	101 (49.8)
Inform about special (regulated) prescription for other drugs, including benzodiazepines	12 (5.9)	31 (15.3)	27 (13.3)	40 (19.7)	93 (45.8)
Informed about possible career restrictions related to psychiatric drug prescription	3 (1.5)	19 (9.4)	43 (21.2)	62 (30.5)	76 (37.4)
Suicidal patients					
Recommend hospitalization even if not clinically necessary	109 (53.7)	58 (28.6)	17 (8.4)	12 (5.9)	7 (3.4)
Increase follow-up	55 (27.1)	48 (23.6)	39 (19.2)	44 (21.7)	17 (8.4)
Share suicidal thoughts with family even if not active and clinically justified	17 (8.4)	46 (22.7)	51 (25.1)	44 (21.7)	45 (22.2)
Consult a peer-level psychiatrist (e.g., fellow resident or colleague)	18 (8.9)	55 (27.1)	54 (26.6)	45 (22.2)	31 (15.3)
Always consider referral for hospitalization.	48 (23.6)	71 (35.0)	52 (25.6)	16 (7.9)	16 (7.9)
Prescribe medication even without clear indication	94 (46.3)	63 (31.0)	21 (10.3)	19 (9.4)	6 (3.0)
Report-related patients					
Inform about potential career impact of reports	6 (3.0)	23 (11.3)	29 (14.3)	57 (28.1)	88 (43.3)
Inform about reports cannot be erased from the database retrospectively	5 (2.5)	15 (7.4)	24 (11.8)	51 (25.1)	108 (53.2)
Request to review past reports	3 (1.5)	2 (1.0)	11 (5.4)	29 (14.3)	158 (77.8)
Avoid judgment in re-evaluation report	70 (34.5)	63 (31.0)	44 (21.7)	14 (6.9)	12 (5.9)
Inform family before mandatory report	3 (1.5)	8 (3.9)	19 (9.4)	66 (32.5)	107 (52.7)
Refrain from judgment in forensic reports	76 (37.4)	64 (31.5)	40 (19.7)	16 (7.9)	7 (3.4)

Regarding medication-related practices, while 36.0% of participants reported that they always documented the explanations they provided about medication side effects, 80.8% indicated that they never obtained signed consent forms outlining these side effects. Furthermore, while 38.4% stated that they routinely ordered laboratory tests despite the absence of a clinical indication, 35.0% reported that they never referred patients to cardiology before initiating mph/atx treatment without a medical necessity. Of the participants, 49.8% reported always informing patients about the special prescription requirement for methylphenidate, and 45.8% did so for other drugs, including benzodiazepines. Additionally, 37.4% stated that they always informed patients about potential career restrictions related to psychiatric drug prescriptions.

In the context of suicidal patients, 3.4% of participants reported always recommending hospitalization even when not clinically necessary, and 3.0% prescribed medication without a clear indication in every patient. The frequency of increasing follow-up for suicidal patients showed considerable variation, with 8.4% reporting this practice for every patient. Sharing suicidal thoughts with families, even when not active and not clinically warranted, was reported by 22.2% for every patient and 21.7% for most patients. Consulting a peer-level psychiatrist in such cases was indicated by 15.3% for every patient and 22.2% for most patients.

Report-related practices were common: approximately half of the participants reported that they informed every patient that medical reports could not be erased from the database (53.2%) and that they informed the family before submitting a mandatory report (52.7%), while 43.3% stated that they always informed patients about the potential career impact of such reports.

### 3.4 Comparison of defensive medicine–related practices between DM and non-DM groups

Participants in the DM group reported higher frequencies of several defensive medicine–related practices across the four assessed domains compared with those in the non-DM group. In the patient assessment domain, the DM group showed significantly higher mean scores for practices such as refusing to perform assessments (p = 0.004) or prescribe medications without a parent/legal guardian (p = 0.004), prescribing medications without the patient (p = 0.015) and basing diagnostic coding on treatment decisions (p = 0.002). Similarly, informing patients about rare but serious side effects (p < 0.001), documenting these discussions (p < 0.001), and obtaining signed consent (p = 0.019) were significantly more common among DM participants. Notably, referrals to cardiology before initiating medications such as methylphenidate or atomoxetine, even in the absence of clinical indication, were also more frequent in this group (p < 0.001).

Regarding the management of suicidal patients, the DM group more often increased follow-up (p = 0.033), consulted peers (p = 0.016), considered hospitalization (p = 0.013), and even prescribed medication without clear indication (p = 0.003). In the context of report-related practices, the DM group was more likely to request a review of previous reports (p = 0.027), and refrain from making evaluative judgments in re-evaluation (p = 0.003) or forensic reports (p = 0.022). Detailed results are presented in **Table 3**.

**Table 3:** Comparison of defensive medicine–related practices between DM group and non-DM group.

Practice item	DM group n:84 mean (SD)	No-DM group n:119 mean (SD)	t/z	P
Patient assessment				
No assessment without parent or legal guardian	4.32 (0.93)	3.85 (1.32)	-2.93	0.004
No psychotherapy without parent or legal guardian	4.40 (1.07)	4.10 (1.29)	-1.76	0.079
No pharmacotherapy without parent or legal guardian	4.70 (0.96)	4.32 (1.29)	-2.88	0.004
Medication not prescribed without patient	3.79 (1.34)	3.31 (1.41)	-2.46	0.015
Diagnostic coding contingent on treatment decision	3.95 (1.16)	3.40 (1.32)	-3.13	0.002
Confirmatory tests sought despite clear clinical impression	3.66 (1.10)	3.49 (1.06)	-1.11	0.268
Patients medication				
Inform about rare but severe adverse effects	3.91 (0.97)	3.30 (1.21)	-3.98	< 0.001
Record that explained about side effects	3.97 (1.20)	3.26 (1.40)	-3.84	< 0.001
Obtain signed consent outlining side effects	1.45 (0.92)	1.15 (0.38)	-2.34	0.019
Inform about suicidality associated with SSRI treatment	2.53 (1.22)	2.31 (1.07)	-1.38	0.159
Routine labs ordered in absence of recent results	4.08 (1.16)	3.68 (1.17)	-2.36	0.019
Referral to cardiology despite absence of indication before mph/atx treatment	2.67 (1.43)	1.90 (1.06)	-4.18	< 0.001
Inform about special (regulated) prescription for mph	4.06 (1.29)	3.82 (1.29)	-1.28	0.202
Inform about special (regulated) prescription for other drugs, including benzodiazepines	4.04 (1.19)	3.69 (1.37)	-1.93	0.055
Informed about possible career restrictions related to psychiatric drug prescription	4.02 (1.06)	3.86 (1.03)	-1.06	0.290
Suicidal patients				
Recommend hospitalization even if not clinically necessary	1.98 (1.24)	1.61 (0.87)	-1.87	0.061
Increase follow-up	2.83 (1.18)	2.44 (1.38)	-2.14	0.033
Share suicidal thoughts with family even if not active and clinically justified	3.44 (1.15)	3.14 (1.32)	-1.65	0.099
Consult a peer-level psychiatrist (e.g., fellow resident or colleague)	3.32 (1.13)	2.90 (1.23)	-2.43	0.016
Always consider referral for hospitalization.	2.65 (1.22)	2.24 (1.08)	-2.51	0.013
Prescribe medication even without clear indication	2.20 (1.27)	1.71 (0.90)	-3.01	0.003
Report-related patients				
Inform about potential career impact of reports	4.13 (1.11)	3.86 (1.14)	-1.63	0.103
Inform about reports cannot be erased from the database retrospectively	4.31 (1.01)	4.10 (1.09)	-1.32	0.188
Request to review past reports	4.78 (0.58)	4.57 (0.84)	-2.21	0.027
Avoid judgment in re-evaluation report	2.48 (1.29)	1.97 (1.00)	-3.04	0.003
Inform family before mandatory report	4.39 (0.83)	4.25 (0.94)	-1.09	0.276
Refrain from judgment in forensic reports	2.29 (1.15)	1.93 (1.02)	-2.31	0.022

### 3.5 Predictor of defensive medicine practice

Among the predictors, a higher weekly number of health committee patients was significantly associated with an increased likelihood of DM practice (B = 0.788, p = 0.017, OR = 2.198, 95% CI [1.148, 4.208]). In terms of specific clinical practices, informing patients about rare but severe adverse effects was a significant positive predictor of DM practice (B = 0.458, p = 0.002, OR = 1.580, 95% CI [1.179, 2.118]). Similarly, referral to cardiology before initiating stimulant or atomoxetine treatment, even in the absence of clinical indication was also significantly predicted of DM practice (B = 0.350, p = 0.010, OR = 1.419, 95% CI [1.089, 1.849]). Other variables, including prescribing medication without clear indication (p = 0.061), diagnostic coding contingent on treatment decision (p = 0.062), and refusing to assess patients without the presence of a parent or guardian (p = 0.069), showed a trend toward significance but did not reach the conventional threshold (**Table 4**).

**Table 4:** Individual predictors of DM.

Predictor variable	B	SE	Wald	p	Exp(B)	95% CI lower	95% CI upper
Weekly number of health committee patients	0.788	0.331	5.653	0.017	2.198	1.148	4.208
No assessment without parent or legal guardian	0.279	0.153	3.316	0.069	1.322	0.979	1.784
Diagnostic coding contingent on treatment decision	0.252	0.135	3.474	0.062	1.286	0.987	1.676
Inform about rare but severe adverse effects	0.458	0.149	9.376	0.002	1.580	1.179	2.118
Referral to cardiology despite absence of indication before mph/atx treatment	0.350	0.135	6.697	0.010	1.419	1.089	1.849
Prescribe medication even without clear indication	0.284	0.152	3.508	0.061	1.329	0.987	1.789

Model: Chi^2^ (6) = 47.775; p <.001, Cox–Snell R^2^ = 0.210; Nagelkerke R^2^ = 0.282.

## 4 Discussion

In this study, we describe self-reported DM practices among child and adolescent psychiatrists, report on their prevalence, and examine their associations with selected sociodemographic and professional characteristics in those who acknowledge practicing defensive medicine. These findings offer one of the first detailed insights into how defensive medicine–related practices manifest in this specialty and highlight several factors that may shape clinicians’ propensity to engage in such practices.

In our study, the prevalence of DM practices among child and adolescent psychiatrists was found to be 41.4%. This rate is lower than those reported in studies conducted with adult psychiatrists, yet it underscores that DM remains a significant phenomenon within the field. For example, Passmore and Leung reported that approximately three-quarters of psychiatrists in the United Kingdom had engaged in at least one defensive practice within the past month, such as unnecessary hospitalization or excessive documentation for legal protection (13). In line with these findings, Reuveni et al. found a prevalence of 62.1% among psychiatrists, and Scognamiglio et al. reported a similar rate of 60.2% using the same methodological approach (2, 27).

One of the major motivations for practicing DM appears to be the fear of litigation (2, 3). Certain litigation-related situations are also more common in adult practice; for example, suicide rates are generally higher among adults compared to children and adolescents (22, 28). Psychiatrists may therefore be sued more frequently in adult psychiatry, which could partly explain this difference; however, this interpretation remains speculative. Indeed, a study evaluating malpractice allegations in psychiatry in Türkiye found that the ages of the cases ranged from 3 to 89 years, with a mean age of 37.35 years (SD = 18.7) (22). This age distribution points to the likelihood that most of the cases were adult patients, although the study did not directly compare malpractice claims between adult and child and adolescent psychiatry.

In Türkiye, within the framework of professional liability insurance for medical malpractice, medical specialties are classified into four risk groups, with group 4 representing the highest risk. Psychiatry, including child and adolescent psychiatry, is placed in group 3 (29). Although our findings indicate a lower prevalence of defensive medicine among child and adolescent psychiatrists compared to adult psychiatry, the existence of legal reporting obligations, the complexity of family dynamics, and the vulnerability of the patient population highlight that defensive medicine remains an important issue that warrants attention in this field.

Research in psychiatry has frequently examined the roles of both age and professional seniority in relation to defensive medical practices. Reuveni et al. reported an inverse relationship between age and acknowledgement of defensive practice, while Morena et al. similarly found that psychiatrists who reported defensive behaviors were younger and had fewer years of seniority compared to their non-defensive counterparts (2, 11). Consistent with these observations, Passmore and Leung demonstrated that less experienced psychiatric trainees were more likely to hospitalize patients or impose excessive monitoring (13). In our study, however, no significant differences were observed between groups in terms of either age or years of seniority. Findings from other specialties have not been consistent with this pattern. For example, a study on obstetricians and gynecologists in Türkiye found that older age was associated with greater defensive practice (30). Findings regarding the role of gender in DM in psychiatry remain mixed. Reuveni et al. reported no overall gender difference in acknowledging DM, whereas Morena et al. found a higher proportion of women among psychiatrists reporting DM (2, 11); studies in other medical specialties have likewise yielded inconsistent findings (8, 30). In our study, no significant difference was observed between the DM and non-DM groups according to gender. Overall, these findings suggest that the associations of age, professional seniority, and gender with DM may vary across clinical contexts and specialties. Nevertheless, further research that takes into account different clinical contexts and additional variables is needed to better clarify these relationships.

Previous research has indicated that physicians managing higher patient volumes are more likely to encounter malpractice claims annually (31). One possible explanation is that a high patient volume may constrain the time available for each encounter, thereby limiting the extent to which patients and their families feel adequately heard and attended to. Inattentiveness and lack of diligence are commonly reported reasons for malpractice claims (22). Such dynamics may be particularly relevant given the reported association between physician–patient communication and malpractice risk. Empirical evidence from primary care demonstrates that physicians without malpractice claims typically allocated longer consultation times, employed facilitative communication techniques, and fostered greater patient engagement, whereas shorter visits were independently associated with increased claim risk (32). In this study, no significant association was found between patient volume and DM. In our context, the defensive medicine–related practices we evaluated largely consisted of time-intensive behaviors, including extended psychoeducation and decisions regarding hospitalization, as opposed to ordering additional tests or procedures. Given that psychiatry is a field in which diagnostic investigations are relatively limited, the absence of a direct relationship between patient load and DM may partly reflect the possibility that some defensive medicine–related practices in this specialty require the allocation of more, rather than less, time to the clinical encounter.

The number of health committee patients, though only marginally significant in the bivariate analysis, emerged as a significant independent predictor of DM in the regression model. While the number of forensic patients showed a trend toward significance in the between-group bivariate analysis, it did not emerge as an independent predictor of DM in the multivariable model. A potential explanation for the salience of this factor in the model lies in the distinct nature of both patient profiles. Unlike a classical physician–patient relationship, these cases often involve structured legal or administrative frameworks (14). They typically mandate procedures such as structured questioning, the administration of supporting assessments like intelligence testing, and the formal reporting of outcomes to official authorities. These mandated, time-intensive procedures may partly account for the observed pattern of DM.

Previous experiences of complaints or involvement in legal proceedings, whether concerning the clinician themselves or their colleagues, have been considered important factors contributing to the practice of DM. In a study among psychiatrists, a colleague’s experience of complaints (56%), legal claims against a colleague (24%), personal experience of complaints (23%), and prior legal claims against oneself (6%) were reported as influential factors in the adoption of defensive practices (13). The rate of malpractice lawsuit involvement in our sample was only 1 percent, which is notably lower than the rates documented in the existing literature (2, 8, 33). Although no significant difference was observed between the groups in terms of malpractice involvement, the very low prevalence of such cases in our sample may have limited our ability to detect an association, warranting further research with greater variability in medico-legal exposure.

Our findings show that defensive practices were commonly observed, extending across all areas of clinical work including patient assessment, medication management, suicide risk management, and documentation. Across the entire sample, clinicians reported that they generally did not initiate clinical assessments, pharmacotherapy, or psychotherapy when a legal guardian or parent was not present. However, between-group comparisons showed that refraining from initiating an assessment and avoiding the initiation of pharmacotherapy were significantly more common among clinicians who identified themselves as practicing DM. These findings should first be considered within the legal and ethical context of assessing and treating minors in Türkiye. The Turkish Regulation on Patient Rights generally requires parental or guardian consent for medical interventions involving minors, while also recognizing circumstances in which this requirement does not apply when the parent or legal guardian is absent or unavailable (34). Furthermore, a recent legal opinion published by the Turkish Society of Dermatology indicated that minors with sufficient decision-making capacity may undergo medical examination without a parent being present, while parental consent remains necessary in certain circumstances (35). It has been reported that parents often prefer psychotherapy over pharmacotherapy due to concerns about potential side effects and perceived risks associated with psychiatric medications (36, 37). Together with the potential long-term implications of psychiatric records in Türkiye discussed above, such parental concerns may provide a possible context for the greater caution regarding assessment and particularly pharmacotherapy observed among clinicians reporting DM when a parent or legal guardian is absent. Notably, although refraining from initiating an assessment differed significantly between the DM and non-DM groups, it did not remain statistically significant as an individual predictor of DM in the logistic regression analysis (p = 0.069). These findings therefore warrant caution in interpreting avoidance of assessment or treatment in the absence of a parent or legal guardian as inherently defensive, as such behaviors may also reflect legal, ethical, and clinical considerations.

The higher frequency of entering diagnostic codes based on treatment decisions among clinicians who identified themselves as practicing DM may be understood in the context of both the inherent subjectivity of psychiatric diagnosis and sociocultural dynamics unique to Türkiye. Psychiatric diagnoses rely heavily on clinical judgment rather than definitive laboratory or imaging markers, which has long been a source of debate regarding their perceived subjectivity. Conditions such as ADHD—where behavioral assessment is central—are particularly susceptible to criticisms of overdiagnosis, potentially increasing clinicians’ concern about being accused of diagnostic errors (38). In Türkiye, this issue may be particularly relevant given that psychiatric diagnoses recorded in childhood can later pose barriers to entry into professions such as police or military service (23, 24). Consequently, requests for the modification or removal of psychiatric diagnoses from medical records may arise in adolescence or early adulthood (25). These considerations may provide a possible context for the greater frequency of treatment-contingent diagnostic coding observed among clinicians reporting DM.

In clinical practice, disclosing all possible adverse effects is neither practical nor necessary, and determining what to share involves considerable uncertainty. This uncertainty is compounded by the lack of clear guidance from legal frameworks such as the professional practice standard, the reasonable-person standard, and patient-specific disclosure (39). Moreover, adverse drug effects are a leading cause of malpractice claims (22). In this context, our finding that the detailed disclosure of serious but rare adverse effects was the strongest predictor of physicians reporting defensive practices is highly significant. In contrast, studies such as those by Reuveni and Morena, conducted among adult psychiatrists, found no significant association between the detailed disclosure of rare adverse effects and DM (2, 11). This discrepancy may be considered in the context of the distinctive features of child and adolescent psychiatry. The heightened responsibility for vulnerable patients, prevalent parental concerns and prejudices about pharmacotherapy, and the essential involvement of families in shared decision-making may be relevant to understanding a more protective approach to disclosing even rare risks. However, as also noted by Reuveni et al., such practices may reflect factors other than defensive motivation and may also represent appropriate clinical practice, including informed consent and shared decision-making (2). Our findings regarding this practice should therefore be interpreted with this distinction in mind.

Routine cardiology consultation for all patients prior to atomoxetine (atx) and methylphenidate (mph) treatment is not recommended in current clinical guidelines in the field of child and adolescent mental health (40, 41). Therefore, the finding in our study that referral to cardiology in the absence of a clear medical indication emerged as a significant predictor of DM represents an expected and internally consistent result. Although the guidelines do not mandate such an evaluation, some clinicians may request cardiac assessment as a “precautionary” measure. The association of this practice with self-reported DM in our study may suggest a more protective approach to treatment decisions, potentially reflecting concerns about medico-legal liability in addition to clinical considerations.

Suicide represents a particularly complex clinical phenomenon in psychiatry and is closely associated with defensive medical practices. Although suicide cannot always be predicted or prevented, psychiatrists are often held to unrealistic expectations, and suicidal outcomes may be perceived as professional failure or negligence. This perception increases concerns about malpractice liability and may lead clinicians to prioritize legal protection over clinical judgment (42). In child and adolescent psychiatry, suicide risk carries additional legal and ethical implications, as adolescents are not considered fully responsible for their actions. Consequently, clinicians are expected to demonstrate heightened vigilance in risk assessment and documentation. In this context, defensive practices such as precautionary hospitalization or obtaining second opinions may arise, sometimes driven more by liability concerns than by clinical necessity, particularly when long-term hospitalization is not feasible (43).

In our study, similar to the findings of Morena et al., clinicians who perceived themselves as practicing DM demonstrated significantly higher rates of hospitalization recommendations, increased follow-up frequency, consultations, and pharmacological treatment initiation (11). This finding indicates that suicidal patients occupy a central role in defensive medical practices in child and adolescent psychiatry, comparable to patterns observed in adult populations. However, as emphasized in previous research, such practices may represent appropriate clinical risk management in some circumstances while also being associated with defensive motivations in others (2). Our findings regarding these suicide-related practices should therefore be interpreted with this distinction in mind. When these practices were further evaluated for their predictive value, only the initiation of pharmacological treatment without a clear clinical indication showed a trend toward statistical significance. Given the generally cautious approach to psychopharmacological decision-making in child and adolescent psychiatry, this trend may suggest that pharmacological treatment decisions in suicidal patients represent a particularly relevant area for defensive considerations.

With respect to report-related practices, more than half of the participants reported routinely informing all patients that health board reports cannot be removed from official records and regarding their potential impact on future career trajectories. This practice did not differ significantly between groups. In Türkiye, structured multidisciplinary service models providing informational, psychosocial, and counseling support for children and families have recently begun to develop through Multidisciplinary Child and Adolescent Mental Health Centers (ÇÖZGEMs), although these centers have not yet been implemented nationwide (44). In this context, the absence of a between-group difference may suggest that routinely providing such information reflects a broader professional responsibility rather than a practice specific to clinicians reporting DM.

In Türkiye, disability health board reports issued by authorized medical boards entitle individuals to a range of social rights, including tax reductions and exemptions, access to social assistance and care services, positive discrimination measures in employment and education, free or discounted transportation, and various other public services and social benefits. The scope and extent of these entitlements vary according to the certified degree of disability (45). Previous research among child and adolescent psychiatrists in Türkiye has highlighted challenges in disability report evaluations, including inconsistencies in assessment, limited support, and substantial workload (17). Given their implications across multiple areas of an individual’s life, report-related decisions may also be particularly sensitive to complaints and medicolegal scrutiny. Indeed, a Turkish study of psychiatric malpractice claims identified erroneous reports as the most frequent reason for complaint and diagnosis–report inconsistency as the most frequent type of fault in cases involving physician error (22). In this context, the greater frequency of some report-related practices among clinicians reporting DM may be related to concerns about potential complaints and medicolegal consequences.

This study has several limitations that should be acknowledged. First, data were collected through an anonymous, self-administered online survey distributed via professional WhatsApp groups and professional networks, with elements of snowball sampling. Participation was entirely voluntary, which may have introduced selection bias, as individuals who were more aware of or interested in the topic of defensive medicine may have been more likely to respond. Because the exact number of eligible child and adolescent psychiatrists who ultimately received the survey invitation could not be determined, a response rate could not be calculated. Current membership data from the national professional association nevertheless provide a useful contextual reference regarding the size and professional composition of the field, although these data postdate the study period. While efforts were made to reach eligible clinicians across Türkiye through nationwide professional networks, the extent to which the sample reflects the national distribution of child and adolescent psychiatrists in terms of age, gender, seniority, academic position, and workplace setting could not be fully assessed. These considerations should be taken into account when generalizing the findings to the broader population of child and adolescent psychiatrists in Türkiye. Additionally, the overall sample size could have been larger to allow for more robust subgroup analyses.

Second, the study relied on self-reported data, which is inherently subject to reporting bias, including the possibility of over- or underestimation of actual defensive practices due to personal interpretation or social desirability. The primary classification of DM was also based on physicians’ self-reported assessment of their own practice. Furthermore, the questionnaire assessed the frequency of DM-related practices rather than directly evaluating the underlying motivation for each behavior. Consequently, some of the behaviors examined may also represent appropriate clinical, ethical, or legally required practice depending on the specific clinical context and should not be interpreted as inherently defensive. Future studies incorporating qualitative methods or directly assessing physicians’ motivations may help clarify this distinction.

Third, the cross-sectional design of the study does not allow conclusions regarding causality or the direction of the observed associations. Accordingly, the professional, clinical, cultural, and medico-legal factors discussed as possible explanations for the findings should be regarded as hypotheses rather than established mechanisms. Longitudinal and more comprehensive study designs are needed to clarify these relationships. Our study focused on sociodemographic and professional factors potentially associated with DM practices; however, future research incorporating psychological variables such as anxiety levels and personality traits may offer a more comprehensive understanding of the underlying causes of DM in child an adolescent psychiatrist.

Despite certain limitations, this study has several notable strengths. To the best of our knowledge, this is the first study to examine DM and DM-related practices in child and adolescent psychiatry. It thereby addresses an important gap in the literature, which has largely focused on adult populations. Moreover, the study adopts a comprehensive approach by investigating a wide range of defensive behaviors spanning clinical decision-making, pharmacological practices, suicide-related management, and report-related processes. This broad perspective allows for a more nuanced understanding of how defensive medicine manifests across different domains of practice. Furthermore, by incorporating both self-perceived defensive medicine tendencies and specific clinical behaviors, the study provides insight into the alignment between clinicians’ perceptions and their practices, thereby strengthening the interpretability of the findings.

## 5 Conclusion

Child and adolescent psychiatry is a field that reflects the highly sensitive and individualized nature of the doctor and patient relationship, as seen in psychiatry, while also involving the care of a particularly vulnerable population, namely children. In this context, it becomes evident that concepts and approaches derived solely from either general psychiatry or pediatrics are often insufficient or difficult to apply directly to this specialty. Therefore, increasing the number of studies specifically focused on this field is essential. Medical practices are also shaped by national health policies and legal frameworks. In Turkey, there is currently no comprehensive mental health law. We believe that studies of this kind can provide valuable insights into clinical practice and help inform future legislation by identifying the key issues that should be addressed in child and adolescent mental health care.

## Data Availability

The raw data supporting the conclusions of this article will be made available by the authors, without undue reservation.
