# Supplementary material for: Defensive medicine–related practices among child and adolescent psychiatrists in Türkiye: prevalence, behavioral patterns, and associated factors
Source: Front Psychiatry. 2026 Sep 22;17:1860742. doi: 10.3389/fpsyt.2026.1860742 (PMC13640142; doi:10.3389/fpsyt.2026.1860742)
Supplement: Supplementary file 1 [file SupplementaryFile1.docx]

**Introductory information provided to participants before completing the survey**

Defensive medicine refers to the attitudes and behaviors that physicians adopt toward patients in order to avoid encountering distressing situations—such as being complained about, receiving disciplinary action from administrators, facing legal proceedings, or being exposed in the media—during or after their professional practice. It has two types: positive defensive medicine, which involves performing unnecessary medical procedures to avoid adverse outcomes, and negative defensive medicine, which refers to physicians avoiding high-risk practices.

Child and adolescent mental health is particularly vulnerable to complaints and litigation due to working with a sensitive population and the need to issue official reports. Moreover, individuals with a history of psychiatric diagnoses may face difficulties in entering certain professions, such as military occupations. These factors may lead child and adolescent psychiatrists to adopt defensive approaches. This study aims to evaluate the defensive medicine practices and approaches of child psychiatrists in our country.

**Defensive Medicine–Related Practices**

**All of the following questions will be answered using the 5-point Likert scale: 5 = In every patient, 4 = In most patients, 3 = In half of the patients, 2 = In a few patients, 1 = In no patient.**

**Patients Evaluation**

1. I do not evaluate the patient without the presence of a parent or legal guardian.
2. I do not initiate psychotherapy for a patient without the presence of a parent or legal guardian.
3. I do not initiate pharmacotherapy for a patient who is not accompanied by a parent or legal guardian.
4. I do not prescribe medication for a patient without seeing the patient in person.
5. If I diagnose a patient but do not plan to start medication, I refrain from entering the diagnostic code.
6. Even when I am clinically confident in the diagnosis, I request supporting tests or scale assessments.

**Patients Medication**

1. I inform patients about serious but rare side effects.
2. I document that I have explained the side effects.
3. I obtain a signed informed consent form listing potential side effects.
4. When starting SSRIs, I inform the patient that the medication may increase suicidal thoughts.
5. If routine blood tests have not been done recently (e.g., within the past year), I request them.
6. Even if not medically necessary, I refer patients to cardiology before starting methylphenidate/atomoxetine.
7. I inform patients that methylphenidate is a special (regulated) prescription medication.
8. For patients to whom I prescribe special prescription medications, I inform them that the medication is a special prescription drug.
9. I inform patients that psychiatric prescriptions could potentially restrict access to certain careers.

**Suicidal Patients**

1. Even if hospitalization is not clinically necessary, I recommend inpatient treatment.
2. Even if I do not think it is clinically necessary, I increase the frequency of follow-ups.
3. Even if suicidal thoughts are not active or not directly related to the patient’s psychiatric condition, I inform the family.
4. I consult with a peer-level psychiatrist (e.g., fellow resident or colleague)
5. I always consider referral for hospitalization.
6. I prescribe medication even if there is no clear indication.

**Report-related Patients**

1. I inform patients seeking reports that the report may affect their future professional life.
2. I inform patients that reports submitted for official purposes cannot be removed retrospectively from the database.
3. For committee report cases, if the patient has previous reports, I request to review them.
4. In re-evaluation report, I avoid giving a definitive opinion.
5. For patients requiring forensic notification, I inform the family before reporting.
6. For patients who meet the criteria for mandatory legal reporting, I inform their family before submitting the notification to the relevant authorities.
