# Supplementary material for: Defensive medicine–related practices among child and adolescent psychiatrists in Türkiye: prevalence, behavioral patterns, and associated factors
Source: Front Psychiatry. 2026 Sep 22;17:1860742. doi: 10.3389/fpsyt.2026.1860742 (PMC13640142; doi:10.3389/fpsyt.2026.1860742)
Supplement: Supplementary file 2 [file SupplementaryFile2.docx]

**Sociodemographic and Professional Characteristics of Participating Physicians (N = 203)**

| **Characteristics** |  | **n (%)** |
| --- | --- | --- |
| Age (years) | 25–30 | 65 (32.0) |
|  | 31–40 | 126 (62.0) |
|  | 41–50 | 11 (5.5) |
|  | >50 | 1 (0.5) |
| Gender | Female | 164 (80.8) |
|  | Male | 39 (19.2) |
| Marital status | Married | 144 (70.9) |
|  | Single | 59 (29.1) |
| Having children | Yes | 83 (40.9) |
|  | No | 120 (59.1) |
| Institution of employment | State hospital | 32 (15.8) |
|  | Training and research hospital | 59 (29.1) |
|  | City hospital | 23 (11.3) |
|  | University hospital | 63 (31.0) |
|  | Private hospital | 1 (0.5) |
|  | Private clinic | 25 (12.3) |
| Department position | Professor | 1 (0.5) |
|  | Associate Professor | 12 (5.9) |
|  | Assistant Professor | 20 (9.9) |
|  | Specialist | 116 (57.1) |
|  | Resident | 54 (26.6) |
| Is there a child psychiatry inpatient unit at your institution? |  |  |
|  | Yes | 39 (19.2) |
|  | No | 164 (80.8) |
| Years of experience as a physician (years) | 0–5 | 56 (27.6) |
|  | 6–10 | 82 (40.4) |
|  | 11–15 | 50 (24.6) |
|  | 16–20 | 11 (5.4) |
|  | >20 | 4 (2.0) |
| Years of experience in child and adolescent psychiatry (years) |  |  |
|  | 0–5 | 80 (39.4) |
|  | 6–10 | 85 (41.8) |
|  | 11–15 | 28 (13.8) |
|  | 16–20 | 6 (3.0) |
|  | >20 | 4 (2.0) |
| Monthly on-call duties in the past year | 0 | 67 (33.0) |
|  | <5 | 49 (24.1) |
|  | 5–10 | 51 (25.1) |
|  | >10 | 36 (17.7) |
| Number of patients seen daily | <10 | 48 (23.6) |
|  | 10–20 | 59 (29.1) |
|  | 21–30 | 77 (37.9) |
|  | >30 | 19 (9.4) |
| Weekly number of medical board patients | 0 | 43 (21.2) |
|  | <10 | 58 (28.6) |
|  | 10–20 | 46 (22.7) |
|  | 21–30 | 21 (10.3) |
|  | >30 | 35 (17.2) |
| Weekly number of forensic patients | 0 | 58 (28.6) |
|  | <5 | 105 (51.7) |
|  | 5–10 | 35 (17.2) |
|  | >10 | 5 (2.5) |
| Perceived professional competence | Very low | 0 (0.0) |
|  | Low | 11 (5.4) |
|  | Moderate | 53 (26.1) |
|  | Good | 111 (54.7) |
|  | Very good | 28 (13.8) |
| Level of professional satisfaction | Very low | 3 (1.5) |
|  | Low | 9 (4.4) |
|  | Moderate | 47 (23.2) |
|  | Good | 95 (46.8) |
|  | Very good | 49 (24.1) |
| Involved in a malpractice lawsuit? | Yes | 2 (1.0) |
|  | No | 201 (99.0) |
| Anyone involved in a malpractice lawsuit? | Yes | 32 (15.8) |
|  | No | 171 (84.2) |
| Reported via formal channels? | Yes | 102 (50.2) |
|  | No | 101 (49.8) |
| Anyone close reported via formal channels? | Yes | 156 (76.8) |
